# Investigating the mechanisms of resveratrol in the treatment of gouty arthritis through the integration of network pharmacology and metabolics

**DOI:** 10.3389/fendo.2024.1438405

**Published:** 2024-10-29

**Authors:** Xiaomin Xu, Donghua Yu, Yu Wang, Xin Jiang, Fang Lu, Shumin Liu

**Affiliations:** Research Institute of Traditional Chinese Medicine, Heilongjiang University of Chinese Medicine, Harbin, China

**Keywords:** metabonomics, network pharmacology, gouty arthritis, resveratrol, NF- kappa B, MAPK, stat3, JAK2

## Abstract

**Objective:**

This study integrates network pharmacology and metabolomics techniques to explore the potential regulatory mechanisms of Res on gouty arthritis (GA).

**Methods:**

Network pharmacology was used to predict the mechanism of Res in regulating GA, and methods such as HE staining, ELISA, immunohistochemistry, Real-time PCR, Western blot, and molecular docking were used to verify the role of NF-κB, MAPK, and JAK/STAT inflammatory signaling pathways in the MSU-induced GA rat model. In addition, non-targeted metabolomics techniques were combined to further investigate the mechanism of Res in treating GA.

**Results:**

The results of network pharmacology showed that Res may exert its therapeutic effects through the NF-κB signaling pathway. Animal experiments demonstrated that in the MSU-induced GA rat model, pathological damage, serum biochemical indicators, and levels of inflammatory factors were significantly increased, and the NF-κB signaling pathway was activated. The intervention of Res significantly reduced pathological damage, serum biochemical indicators, levels of inflammatory factors, and the activation of NF-κB, MAPK, and JAK/STAT signaling pathways in the model rats. Metabolomics results showed that Res could improve the metabolic trajectory deviations in serum and joint fluid of GA model rats. Through related metabolic pathway analysis, the most affected metabolic pathways were found to be Sphingolipid metabolism, Glycerophospholipid metabolism, Phenylalanine, tyrosine and tryptophan biosynthesis, Pantothenate and CoA, Citrate cycle (TCA cycle), and Arachidonic acid metabolism.

**Conclusion:**

Resveratrol can regulate the biosynthetic pathways of arachidonic acid, phenylalanine, tyrosine, and tryptophan, pantothenic acid and CoA biosynthesis pathways, TCA cycle, and other metabolic pathways, thereby regulating the NF-κB, MAPK, and JAK/STAT3 signaling pathways, and inhibiting the acute inflammatory response during GA attacks, showing characteristics of multi-pathway and multi-target action.

## 1 Introduction

Gout is considered a common inflammatory joint disease(inflammatory arthropathy) characterized by hyperuricemia and the deposition of Monosodium urate (MSU) crystals and subsequent crystal-related lesions and inflammation. High levels of serum uric acid can lead to intense inflammatory processes with pain and the formation of tophi, exacerbating gouty inflammation (1). Among them, the joints are frequently affected by gout attacks, ultimately leading to acute gouty arthritis (GA) characterized by recurrent joint swelling and pain (2, 3). Although not life-threatening, GA can significantly reduce work efficiency and quality of life, imposing a heavy burden on the healthcare system. Currently, the first-line drugs for the clinical treatment of gout include nonsteroidal anti-inflammatory drugs, colchicine, and glucocorticoids. While these drugs have shown good efficacy in managing gout attacks, their clinical use is limited due to the development of resistance, endogenous hormone suppression, gastrointestinal irritation, and other adverse reactions (4–6). Therefore, it is necessary to search for drugs with minimal toxic side effects, high efficiency, and mild action.

Resveratrol (Res) is a non-flavonoid polyphenolic compound containing stilbene structures, which is widely found in plants in nature. Previous studies have shown that Res exhibits antioxidant, antibacterial, anti-inflammatory, and antiplatelet aggregation effects (7). It has been demonstrated to significantly reduce uric acid levels in mice with hyperuricemia (8) and improve joint swelling and attenuate the incidence of GA in GA mice (9). However, its specific mechanism of action remains unclear. Therefore, this study aims to investigate the specific mechanism of action of Res in GA by constructing a rat model of GA and utilizing network pharmacology combined with metabolomics technology.

Network pharmacology, based on publicly available and published data, can establish specific drug action mechanism network prediction models, decipher drug action mechanisms, and predict drug targets (10). Network pharmacology utilizes big data analysis and network visualization in drug research, integrating bioinformatics, chemical information, and systems biology. It explores the relationship between target proteins and diseases from a holistic perspective, revealing the overall effects of target proteins on disease networks.

Metabolomics is the detection, identification, and quantification of small-molecule compounds involved in metabolism. Its most widely used application is the identification of biomarkers for diagnosis and prediction of diseases, which has great potential in elucidating disease mechanisms (11). In a series of metabolic diseases such as gout and gouty arthritis, metabolomics approaches have identified potential biomarkers that can be used as disease indicators and new therapeutic targets (12, 13). Therefore, this study integrates network pharmacology and metabolomics technology to investigate the mechanism of Res in regulating GA, opening up new avenues for the study of the pathological mechanisms of GA.

## 2 Experimental materials

### 2.1 Experimental animals

Clean-grade (SPF grade) male SD rats, 8 weeks old, weighing (200 ± 20)g, were provided by the Experimental Animal Center of Heilongjiang University of Chinese Medicine, license number: SCXK (Hei) 2018-007, housed at a temperature of 20-25°C and relative humidity of 40%-60%. The experimental animals were given standard feed and free access to water, and the study was approved by the Ethics Committee of Heilongjiang University of Chinese Medicine, approval number DXLL2019081601.

### 2.2 Experimental reagents

Resveratrol (PubChem CID:445154,Sigma, USA, batch number: S30630); Colchicine (Xishuangbanna Pharmaceutical Co., Ltd., batch number: H53021369); Sodium urate (Shanghai Aladdin Biochemical Technology Co., Ltd., batch number: J2122579); Tween 80 (Tianjin Fuyu Fine Chemical Co., Ltd.); Sodium pentobarbital (Tianjin Damao Chemical Reagent Factory); 4% paraformaldehyde tissue fixative (Shanghai Biyuntian Biotechnology Co., Ltd.); Hematoxylin-eosin staining solution (Zhuhai Beso Biotechnology Co., Ltd.); Glacial acetic acid (Shanghai Pharmaceutical Group Chemical Reagent Co., Ltd.); Rat serum IL-1β detection kit (Nanjing Jiancheng Bioengineering Institute, batch number: 20201208); Rat serum IL-8 detection kit (Nanjing Jiancheng Bioengineering Institute, batch number: 20201113); Rat serum TNF-α detection kit (Nanjing Jiancheng Bioengineering Institute, batch number: 20211025); Rat serum MPO detection kit (Nanjing Jiancheng Bioengineering Institute, batch number: 20240723); Rat serum IL-6 detection kit (Nanjing Jiancheng Bioengineering Institute, batch number: 20240820); Rat serum IL-12 detection kit (Nanjing Jiancheng Bioengineering Institute, batch number: 20240820); Rat serum IL-18 detection kit (Nanjing Jiancheng Bioengineering Institute, batch number: 20240820);Methanol (chromatographic grade, Dikma Technologies, 50102); Leu-enkephalin (Sigma-Aldrich, L9133);Actin(Servicebio,GB15003);p65(Servicebio,GB11997);p-p65(Servicebio,GB113882);IκBα(Servicebio,GB13212-1);p-IκBα(Servicebio,GB15212);p38(Servicebio,GB114685);p-p38(Servicebio,GB153380);STAT3(Servicebio,GB11176);p-STAT3(Servicebio,GB150001);JAK2(Servicebio,GB11325);p-JAK2(Servicebio,GB114585).

## 3 Experimental methods

### 3.1 Experimental methods

The SMILES code corresponding to resveratrol was queried using the PubChem database (http://pubchem.ncbi.nlm.nih.gov), and target prediction was performed using the SwissTargetPrediction database (http://www.swisstargetprediction.ch/). The GeneCards database (https://www.genecards.org/) was used to retrieve information on “Gouty arthritis”, and the Uniprot database was employed to standardize gene and protein names. And veeny2.1.0 (http://www.liuxiaoyuyuan.cn/) was used to take the intersection of disease target and drug target as a predictive target for the threapeutic effects of the drug on the disease. The obtained information on the interaction between resveratrol and gouty arthritis disease targets was imported into the STRING database (https://cn.string-db.org/) to identify potential connections between the targets. Cytoscape 3.7.2 software was utilized to visualize the protein-protein interaction analysis results from the STRING database, thereby constructing a protein-protein interaction network. The DAVID platform (https://david.ncifcrf.gov/) was employed for GO functional annotation enrichment analysis and KEGG pathway analysis of the common targets, and visualised using Wei Sheng Xin (https://www.bioinformatics.com.cn/) (14). The target proteins corresponding to resveratrol were directly mapped onto pathways, with the enriched pathways of drug targets representing the potential pathways for the drug’s therapeutic effects. Finally, a bubble chart was generated for visualization.

### 3.2 Animal grouping, modeling, and administration

48 clean-grade (SPF grade) male SD rats, 8 weeks old, weighing (200 ± 20) g, were randomly divided into normal control group, model group, positive control group, and high, medium, and low dose groups of resveratrol, with 8 rats in each group. After one week of adaptation, except for the blank group, the rats in each group were injected with 0.2 mL of uric acid sodium solution (concentration: 25 mg/mL) into the joint cavity on the posterior side of the right hind ankle joint, inserted at an angle of 30-40° towards the Achilles tendon, with the bulging of the joint capsule on the opposite side as the injection standard. The blank group received physiological saline injection into the abdominal cavity and joint cavity at the same site. After modeling, the body weight was measured and the corresponding dose was calculated. The positive control group was orally administered with 0.3 mg/kg colchicine solution for 7 days; the resveratrol treatment groups were given high (1000 mg/kg), medium (500 mg/kg), and low dose (250 mg/kg) of Polygonum cuspidatum compound aqueous solution for 7 days.

### 3.3 Sample collection and processing

After drug administration, 3% pentobarbital sodium-anesthetized rats were used for microdialysis to collect dialysate from the ankle joint tissues of each group of rats. The concentrated residue was re-dissolved in methanol, centrifuged at 12000rpm, and then 100μL of the supernatant was taken and placed in the sample vial for UPLC-MS/MS detection. Blood was collected from the abdominal aorta, allowed to clot at room temperature for 1 hour, centrifuged at 3500r.min-1, 4°C for 15 minutes to separate serum for ELISA and UPLC-MS/MS detection. After blood collection, ankle joint tissues of rats were fixed in 10% paraformaldehyde, and the remaining tissues were stored at -80°C.

### 3.4 Histopathological and biochemical analysis of rat ankle joint tissues

Rat ankle synovial tissues from each group were collected, fixed, and embedded in paraffin following routine procedures. 4 μm thick sections were cut and subjected to deparaffinization in xylene, dehydration in graded ethanol, and then stained and fixed according to the experimental procedure.

After serum separation, 50 μl of serum was taken to measure the levels of serum uric acid (SUA), serum creatinine (SCr), and blood urea nitrogen (BUN) using an automated biochemical analyzer. Additionally, 50 μl of serum was used to measure the levels of myeloperoxidase (MPO),interleukin-6 (IL-6),interleukin-8 (IL-8),interleukin-1β (IL-1β),interleukin-18(IL-18),interleukin-12(IL-12) and tumor necrosis factor-α (TNF-α) following the instructions provided with the respective assay kits. The data were calculated using ELISAcalc (Enzyme-linked immunosorbent assay) software, and a logistic curve (four parameters) was used for model fitting.

### 3.5 Detection of toll-like receptor 4/myeloid differentiation factor 88/nuclear factor kappa-B mRNA expression levels in rat ankle joint tissues using real-time quantitative polymerase chain reaction

Total RNA was extracted from rat ankle joints using Trizol, followed by reverse transcription into cDNA using a kit. Amplification was performed with fluorescent dyes, and PCR amplification reaction system was added with GAPDH as the internal reference gene, with 3 replicates for each target gene sample. Reaction conditions: pre-denaturation at 95°C for 30s, denaturation at 95°C for 5s, annealing at 60°C for 31s, 40 cycles, amplification at 95°C for 15s. The relative expression levels of TLR4, MyD88, and NF-κB mRNA in each group were detected using the 2-ΔΔCt method, with primer sequences shown in **Table 1**.

**Table 1:** Primer sequences.

Primer	Sequence (5'-3’)	Length/bp
GAPDH	F5'-TTTGAGGGTGCAGCGAACTT-3'	142
	R5'- ACAGCAACAGGGTGGTGGAC-3'	
TLR4	F5'- CCGCTCTGGCATCATCTTCA-3'	107
	R5'- CCCACTCGAGGTAGGTGTTTCTG-3'	
MyD88	F5'- TATACCAACCCTTGCACCAAGTC-3'	121
	R5'- TCAGGCTCCAAGTCAGCTCATC-3'	
NF-κB	F5'- TGACGGGAGGGGAAGAAATC-3'	91

### 3.6 Immunohistochemical protein expression detection of TLR4, MyD88, and NF-κB in rat ankle joint tissues in gouty arthritis rats

Ankle joint tissues were collected and baked at 60°C for 30-60 minutes, followed by deparaffinization of the sections to water. Sections were placed in xylene for 10 minutes, twice. Then, they were sequentially placed in 100%, 95%, 85%, and 75% ethanol for 5-10 minutes at each level. After that, the sections were washed with distilled water for 5 minutes. Antigen retrieval was performed by immersing the sections in citrate buffer (pH 6.0) and heating them in a microwave oven on high power for 8 minutes, followed by cooling to room temperature. After cooling, the sections were washed with 0.01 M PBS (pH 7.2-7.6) for 3 minutes, three times. A 3% hydrogen peroxide solution was added to the sections to deactivate endogenous enzymes at room temperature for 10 minutes. The sections were washed with PBS for 3 minutes, three times. Serum blocking was performed by adding 5% goat serum to the sections and incubating at room temperature for 20 minutes. The sections were then washed with PBS for 3 minutes, three times. The appropriate dilution of primary antibody was added dropwise, and for the control group, an equal amount of PBS was added. The sections were incubated overnight at 4°C. For the secondary antibody incubation, the slides were taken out of the refrigerator and allowed to warm up in a 37°C incubator for 30 minutes. They were then washed with PBS for 5 minutes, three times. 50-100 μL of secondary antibody was added dropwise, and the slides were incubated at 37°C for 30 minutes. After that, they were washed with PBS for 5 minutes, three times. HRP-conjugated streptavidin was added dropwise, and the slides were incubated at 37°C for 20 minutes. For DAB staining, the slides were washed with PBS for 5 minutes, three times, and 50-100 μL of prepared DAB working solution was added dropwise. The slides were incubated at room temperature for 1-5 minutes, with reaction time controlled under microscopy. After staining, the slides were washed with distilled water, immersed in hematoxylin for 1-3 minutes, washed with distilled water again, and then returned to a blue color with PBS. Dehydration was performed with graded alcohols (60-100%) for 5 minutes each. Clearing was done by placing the slides in xylene for 10 minutes, twice. Finally, the slides were mounted with neutral gum for microscopic observation.

### 3.7 Molecular docking

The 3D structure of the active ingredient was downloaded from the PubChem database and saved in mol2 format. The PDB format files of the core targets were downloaded from the PDB database (http://www.rcsb.org/). The protein was preprocessed using molecular docking software, including repair of protein amino acid residues, protonation of 3D hydrogens, charge operation, force field parameter setting, removal of water molecules, and energy minimization. Then, the ligands were energy-minimized. Finally, the docking scores between each compound and the receptor were obtained using the ASE scoring function based on the interaction energy between the protein and ligand. The lower the docking score, the more stable the binding between the ligand and receptor. The docking parameters are shown in **Table 2**.

**Table 2:** Docking parameters between res and target proteins.

Target	center_x	center_y	center_z
TLR4	13.988	0.115	8.276
MyD88	0.405	2.295	-0.38
NF-κB	62.502	11.629	37.997

### 3.8 Western blot analysis

Add pre-cooled lysis buffer (lysis buffer: protease and phosphatase inhibitors = 200:1) to the ankle tissue precipitate, mix well, and place on ice for 30 minutes. Centrifuge at 4°C, 12000rpm for 20 minutes. Measure the protein concentration of the sample using a BCA assay kit. Separate the sample containing 30μg of protein on a 10%-12% SDS-PAGE gel, and transfer to a PVDF membrane after electrophoresis. Block the membrane with 5% skim milk at room temperature for 1 hour. Wash off the residual milk powder on the membrane with TBST. Then incubate with the following primary antibody: p65, p-p65, IκBα, p-IκBα, p38, p-p38, STAT3, p-STAT3, JAK2, p-JAK2 (1:1000). After incubation with an HRP-conjugated secondary antibody (1:5000) at room temperature for 60 minutes, wash the membrane with TBST three times for 5 minutes each. Add the mixed ECL luminescent solution and expose it in a chemiluminescence imaging system. Actin is used as an internal reference (Actin, 1:3000), and the band grayscale values are analyzed using ImageJ software.

### 3.9 Untargeted UPLC-Q-TOF MS analysis

UPLC conditions: A Waters ultra-performance liquid chromatography system coupled with a quadrupole time-of-flight mass spectrometer (UPLC-Q-TOF/MS) was used. The column used was a BEH C18 column (2.1mm×100mm, 1.7μm i.d.; Waters Corp., Milford, USA). The mobile phase A consisted of acetonitrile with 0.05% formic acid, and the mobile phase B consisted of water with 0.05% formic acid. The gradient elution program was as follows: 0-8 minutes: 98%-60% B, 8-10 minutes: 60%-2% B, 10-13 minutes: 2%-0% B, 13-14 minutes: 0%-98% B, 14-17 minutes: 98%-98% B. The flow rate was 0.40 mL/min, injection volume was 2 μL, and column temperature was 40°C. The sample compartment temperature was maintained at 5°C.

Mass spectrometry conditions: Full-wavelength scanning was performed using a photodiode array detector, and the UV detector effluent was directly introduced into the mass spectrometry system without splitting. Electrospray ionization (ESI) source was used in both positive and negative ion modes. The lock mass NC was set to 2.0 μg·L-1 with a flow rate of 40 μL·min-1. The desolvation gas temperature was set at 350°C with a flow rate of 750.0 L/h, and the ion source temperature was set at 110°C. The cone gas flow rate was set at 20 L/h. The capillary voltage was set at 1300.0V for positive ions and 1500.0V for negative ions. The sample cone voltage was set at 60.0V for positive ions and 70.0V for negative ions. LockSprayTM calibration system was used for online mass calibration using leucine enkephalin, and the data acquisition range was m/z 100-1500 Da, using full scan mode.

The raw data processed by UPLC-Q-TOF-MS were aligned, peak matched, denoised, and normalized. ProgenesisQI software and EZinfo2.0 software (MarkerLynx1.4 workstation) were used for serum and ankle tissue data analysis. Unsupervised statistical analysis was performed using principal component analysis (PCA), and supervised partial least squares-discriminant analysis (PLS-DA) was used to group the test samples, and corresponding score plots were obtained. To avoid overfitting of the PLS-DA model, default cross-validation was performed using SIMCA-P software 12.0, and the data were tested using 200 random permutations. The Scores plot graph was used to depict the degree of inter-group dispersion, which was used to determine the similarity of the trajectories of the rat serum and ankle tissue samples in each group and the presence of clustering effects. The S-plot graph was used to show the intergroup changes in potential biomarkers. Variables with a variable importance in projection (VIP) value >1 and P<0.05, and a fold change (FC)>1.2 were used as screening criteria to identify potential characteristic biomarkers. Enrichment analysis and network construction of relevant metabolic pathways were performed using databases such as the Human Metabolome Database (HMDB), Kyoto Encyclopedia of Genes and Genomes (KEGG), and MetaboAnalyst (http://www.metaboanalyst.ca/) to provide more biological information on the physiological and pathological states of differential metabolites, and to identify differential metabolites and metabolic pathways related to the study of diseases.

### 3.10 Statistical analysis

SPSS 26.0 software was used for statistical analysis. Experimental data were expressed as mean ± standard deviation (`X ± S). One-way analysis of variance (ANOVA) was used for intergroup data comparison. A P value <0.05 was considered statistically significant, and P<0.01 was considered highly significant.

## 4 Experimental results

### 4.1 Network pharmacology analysis

**Figure 1:**
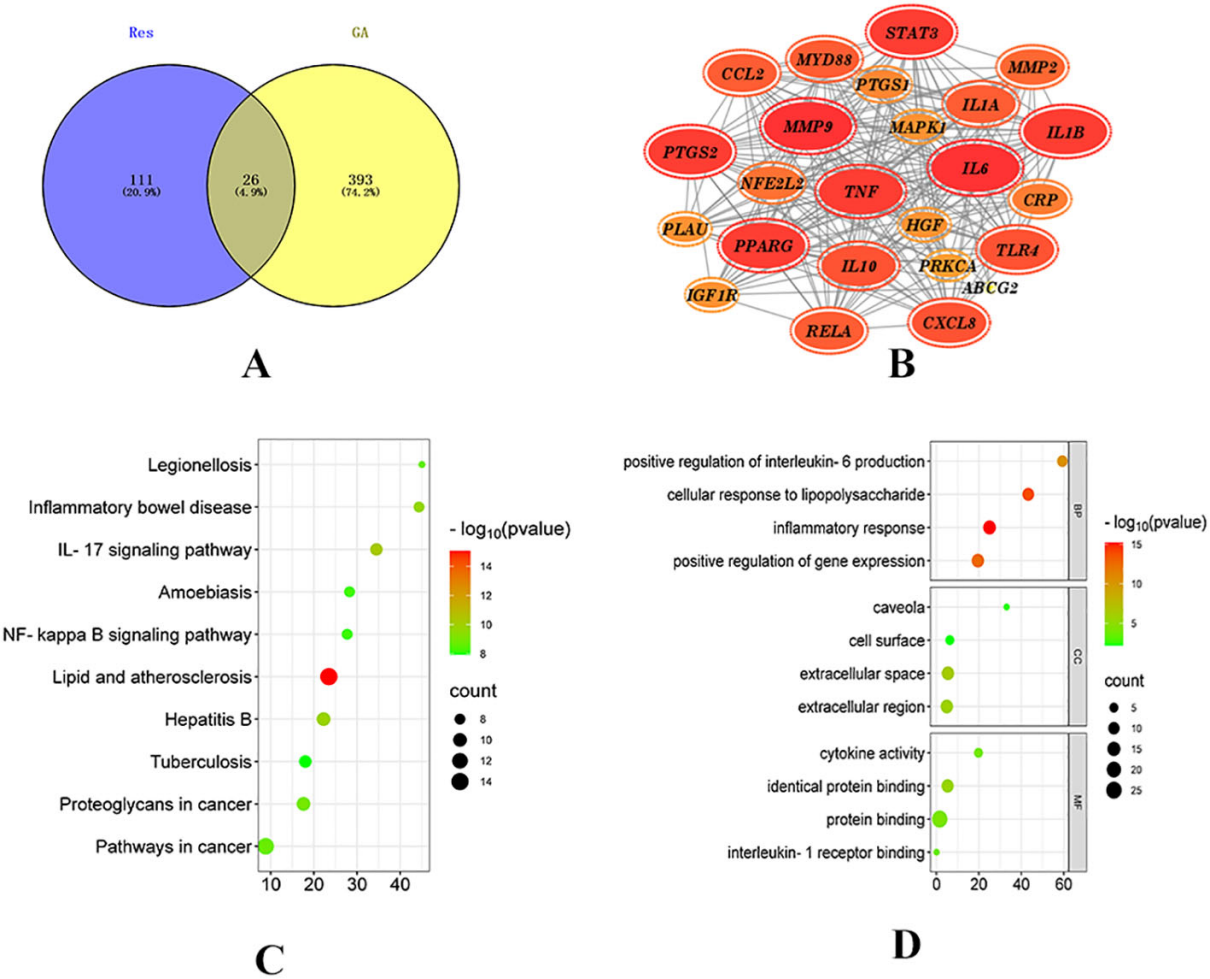
Network pharmacology analysis of Resveratrol on GA. **(A)** Intersection of drug targets and disease targets (15); **(B)** PPI network diagram; **(C)** GO enrichment analysis diagram; **(D)** KEGG pathway diagram (14).

Network pharmacology was employed to explore the potential targets and signaling pathways involved in the treatment of GA by Resveratrol (Res). By conducting a screening analysis, a total of 132 drug targets and 419 disease targets were identified. Among them, 26 targets were found to be common to both Res and GA, suggesting that they represent potential targets of Res in combating GA (**Figure 1A**). Subsequently, the overlapping targets were inputted into the STRING database, with the species specified as “Homo sapiens”, to conduct protein-protein interaction analysis. The resulting network, consisting of 24 nodes and 216 edges, represents the interactions among the target proteins. In the network, each node represents a specific target protein, while each edge represents the interaction between two proteins. The number of edges indicates the level of significance of the interactions. To visualize the important targets in the network, a core target map was generated based on the frequency of protein interactions using Cytoscape software (**Figure 1B**). In addition, enrichment analysis of disease pathways was performed using the DAVID and KEGG databases.

The Gene Ontology (GO) database describes the biological processes (BP), molecular functions (MF), and cellular components (CC) (16) involving gene products, such as proteins or RNAs. GO enrichment analysis, with a significance threshold set at p < 0.05, resulted in the identification of 229 BP, 26 MF, and 10 CC enriched terms. The top 20 ranked biological processes, cellular components, and molecular functions were visualized using a bubble plot (**Figure 1C**).

The KEGG (Kyoto Encyclopedia of Genes and Genomes) database provides annotations for cellular and organismal functions, including pathway maps, BRITE hierarchical structures, and KEGG modules (17). By mapping the target proteins associated with Res to KEGG pathways, a total of 109 enriched pathways were obtained, with a significance threshold set at p < 0.05. A bubble plot was created to display the top 20 enriched pathways based on measures such as fold enrichment and p-value (**Figure 1D**). Among the top 10 pathways, 8 pathways related to diseases, such as Legionellosis, Inflammatory bowel disease, Hepatitis B, and Atherosclerosis, were excluded. Two signaling pathways, namely the IL-17 signaling pathway and the NF-kappaB signaling pathway, remained. The NF-kappaB signaling pathway is part of the Toll-like receptor signaling pathway subcategory. Toll-like receptors (TLRs), a type of transmembrane protein consisting of extracellular, intracellular, and transmembrane regions, are involved in processes such as cytokine secretion and recognition of disease-associated molecular patterns, thereby regulating immune responses. Proteins within this receptor family are closely associated with cell apoptosis and inflammatory responses (18). Recent studies have demonstrated that (19) Toll-like receptors (TLRs), particularly TLR4, which is a type I transmembrane protein and an effective activator of inflammatory responses, play a role in the development of gouty arthritis. Upon stimulation by monosodium urate (MSU), TLRs are activated, and the subsequent recruitment of proteins such as myeloid differentiation factor 88 (MyD88) and interleukin-1 receptor-associated kinases (IRAKs) leads to the activation of nuclear factor kappa-B (NF-κB) by IKB kinases. Activated NF-κB enters the nucleus and initiates the transcription of inflammatory factors, thereby promoting the production of precursor molecules such as NLRP3, IL-18, and IL-1β (20). Moreover, studies have confirmed that gout attacks are mediated by the activation of the toll-like receptor 4 (TLR4)/MyD88/NF-κB signaling pathway in response to urate salts, highlighting the involvement of this pathway in regulating immunity and inflammatory responses during the development of gouty arthritis (21, 22).

### 4.2 Histopathological observation of rat ankle synovial tissue

**Figure 2:**
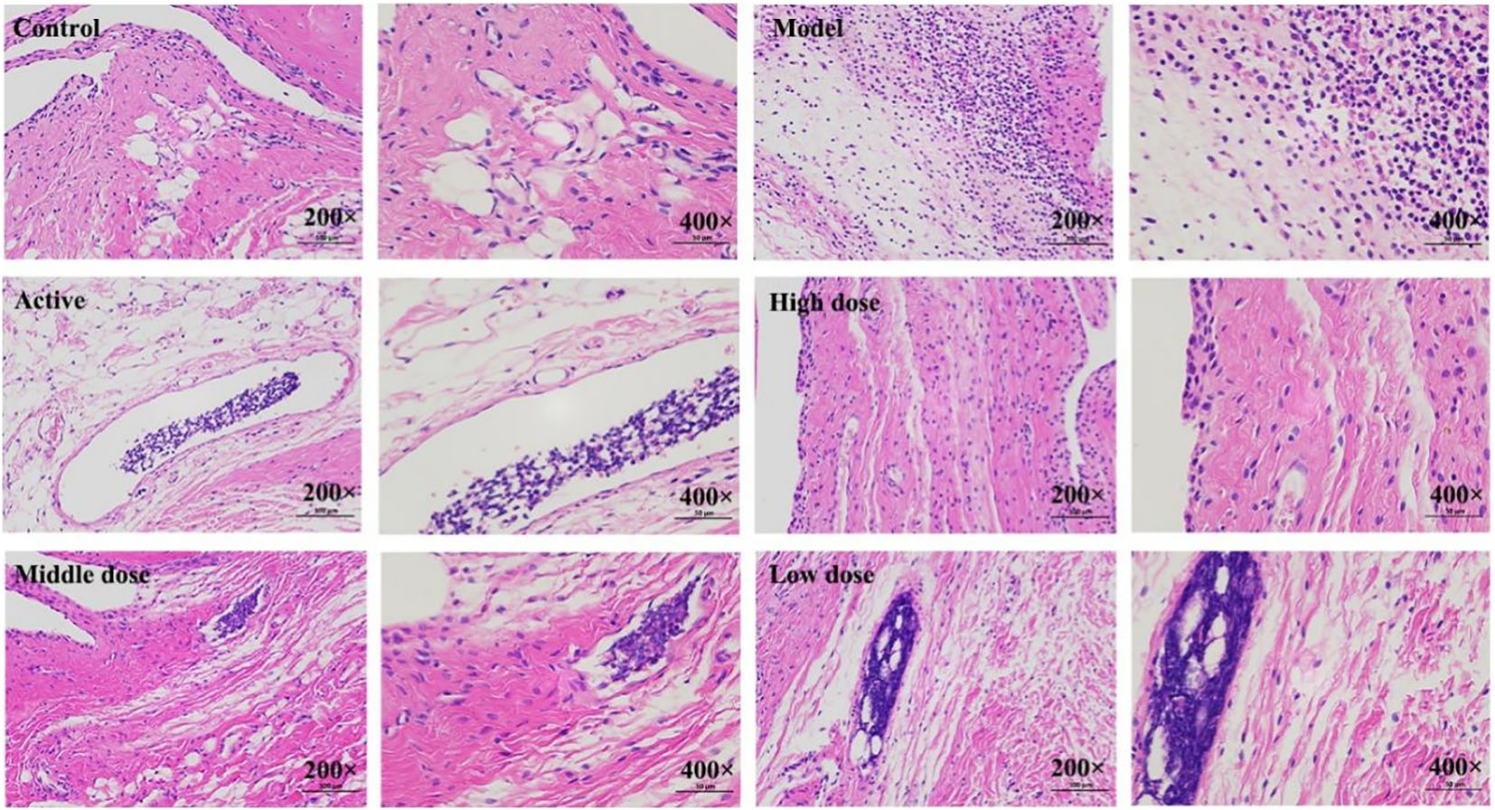
Histopathological observation of rat ankle joint tissues. Control: blank group; Model: model group; Active: positive control group; High dose: high dose of Resveratrol group; Middle dose: medium dose of Resveratrol group; Low dose: low dose of Resveratrol group.

The results are shown in (**Figure 2**). The pathological observations of ankle joint tissues in each group of rats are as follows: in the blank group, the synovial membrane structure is intact, with abundant and orderly arranged collagen fibers, and a small amount of lymphocytes and neutrophils infiltration can be seen. Compared with the blank group, in the model group, severe lymphocyte and neutrophil infiltration can be seen in the synovium on one side of the joint, accompanied by edema in multiple areas, loose arrangement of connective tissue, and a large amount of necrotic cell fragments can be observed; a large amount of eosinophilic exudate can be seen in the joint cavity. Compared with the model group, in the positive control group, there are localized lymphocyte and neutrophil infiltrations in the synovium on one side of the joint; in the high-dose Res group, edema can be seen in the synovium on one side of the joint, with loose arrangement of connective tissue and scattered lymphocyte infiltration; in the medium-dose Res group, localized lymphocyte infiltration can be seen in the synovium on one side of the joint; in the low-dose Res group, there is mild connective tissue hyperplasia in the synovium on one side of the joint, accompanied by a small amount of lymphocyte infiltration. The quantitative analysis of neutrophils and lymphocytes is shown in (**Table 3**). These results suggest that Res can significantly alleviate the degree of pathological damage in the joint tissues of rats with GA and has a significant anti-inflammatory effect.

**Table 3:** Quantitative analysis of Neutrophils and Lymphocytes.

Grouping	Number of neutrophils	Number of lymphocytes
Control	3	1
Model	124	30
Active	3	7
High-dose group	1	0
Middle-dose group	1	2
Low-dose group	5	0

### 4.3 Measurement of serum levels of SUA, SCr, BUN, MPO and ROS in rats

**Figure 3:**
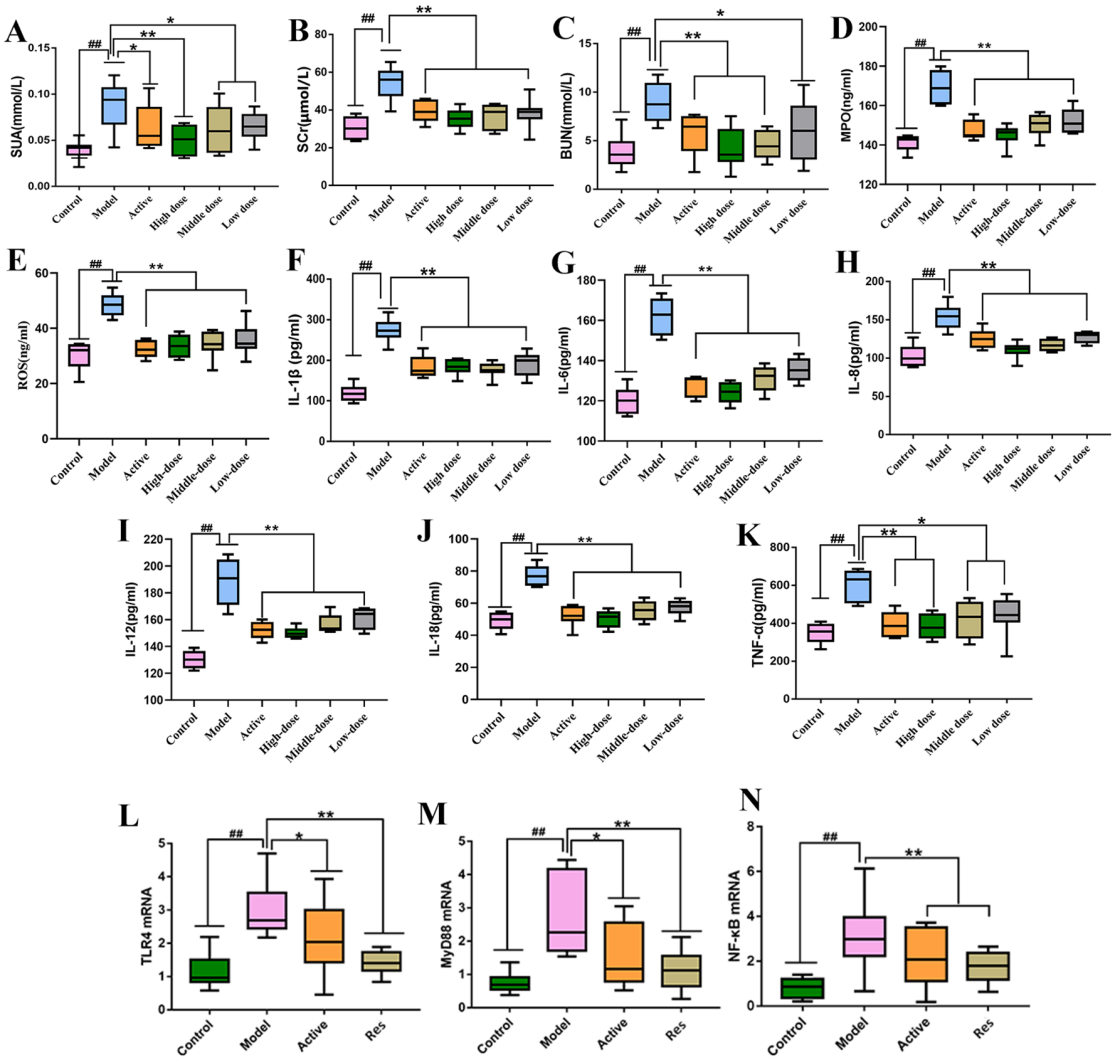
Impact of Resveratrol on Serum Biochemical Markers and TLR4, MyD88, NF-κB mRNA in Rat Ankle Synovial Tissue. **(A)** Impact of Resveratrol on Serum SUA in GA Rats; **(B)** Impact of Resveratrol on Serum SCr in GA Rats; **(C)** Impact of Resveratrol on Serum BUN in GA Rats; **(D)** Impact of Resveratrol on Serum MPO in GA Rats; **(E)** Impact of Resveratrol on Serum ROS in GA Rats; **(F)** Impact of Resveratrol on Serum IL-1β in GA Rats; **(G)** Impact of Resveratrol on Serum IL-6 in GA Rats; **(H)** Impact of Resveratrol on Serum IL-8 in GA Rats; **(I)** Impact of Resveratrol on Serum IL-12 in GA Rats; **(J)** Impact of Resveratrol on Serum IL-18 in GA Rats; **(K)** Impact of Resveratrol on Serum TNF-α in GA Rats; **(L)** Impact of Resveratrol on TLR4 mRNA in GA Rat Ankle Synovial Tissue; **(M)** Impact of Resveratrol on MyD88 mRNA in GA Rat Ankle Synovial Tissue; **(N)** Impact of Resveratrol on NF-κB mRNA in GA Rat Ankle Synovial Tissue. Compared to the blank control group, ^#^p<0.05, ^##^p<0.01; Compared to the model group, *p<0.05, **p<0.01.

Serum levels of SUA, SCr, BUN, MPO and ROS were measured in rats from each group. The results are shown in (**Figures 3A–E**). Compared to the blank control group, the model group exhibited significantly elevated levels of serum SUA, SCr, BUN and MPO (*P < 0.01*). In contrast, the levels of these parameters were significantly reduced in the treatment groups compared to the model group, with varying degrees of decrease (*P < 0.01*, *P < 0.05*). Among them, the high-dose combination group showed the most pronounced effects.

### 4.4 Measurement of serum levels of IL-1β,IL-6, IL-8, IL-12,IL-18 and TNF-α in rats

Serum levels of IL-1β,IL-6, IL-8, IL-12,IL-18 and TNF-α were measured in rats from each group. The results are shown in (**Figures 3F–K**). Compared to the blank control group, the model group exhibited significantly elevated levels of serum IL-1β, IL-8, IL-6,IL-12,IL-18 and TNF-α (*P < 0.01*). In contrast, the levels of these parameters were significantly reduced in the treatment groups compared to the model group, with varying degrees of decrease (*P < 0.01*, *P < 0.05*). Among them, the high-dose combination group showed the most pronounced effects.

### 4.5 Measurement of TLR4, MyD88, and NF-κB mRNA levels in rat ankle synovial tissue by real-time PCR

The levels of TLR4, MyD88, and NF-κB mRNA in rat ankle synovial tissue were measured in each group. The results showed that compared to the normal control group, the model group exhibited significantly elevated levels of TLR4, MyD88, and NF-κB mRNA in ankle synovial tissue (*P < 0.01*). In comparison to the model group, both the positive control group and the treatment groups showed significant reductions in TLR4, MyD88, and NF-κB mRNA levels (*P < 0.01*, *P < 0.05*), with the positive control group showing a lower downregulation trend compared to the Resveratrol treatment groups (**Figures 3L–N**).

### 4.6 Immunohistochemical expression of TLR4, MyD88, and NF-κB Proteins in ankle synovial tissue

**Figure 4:**
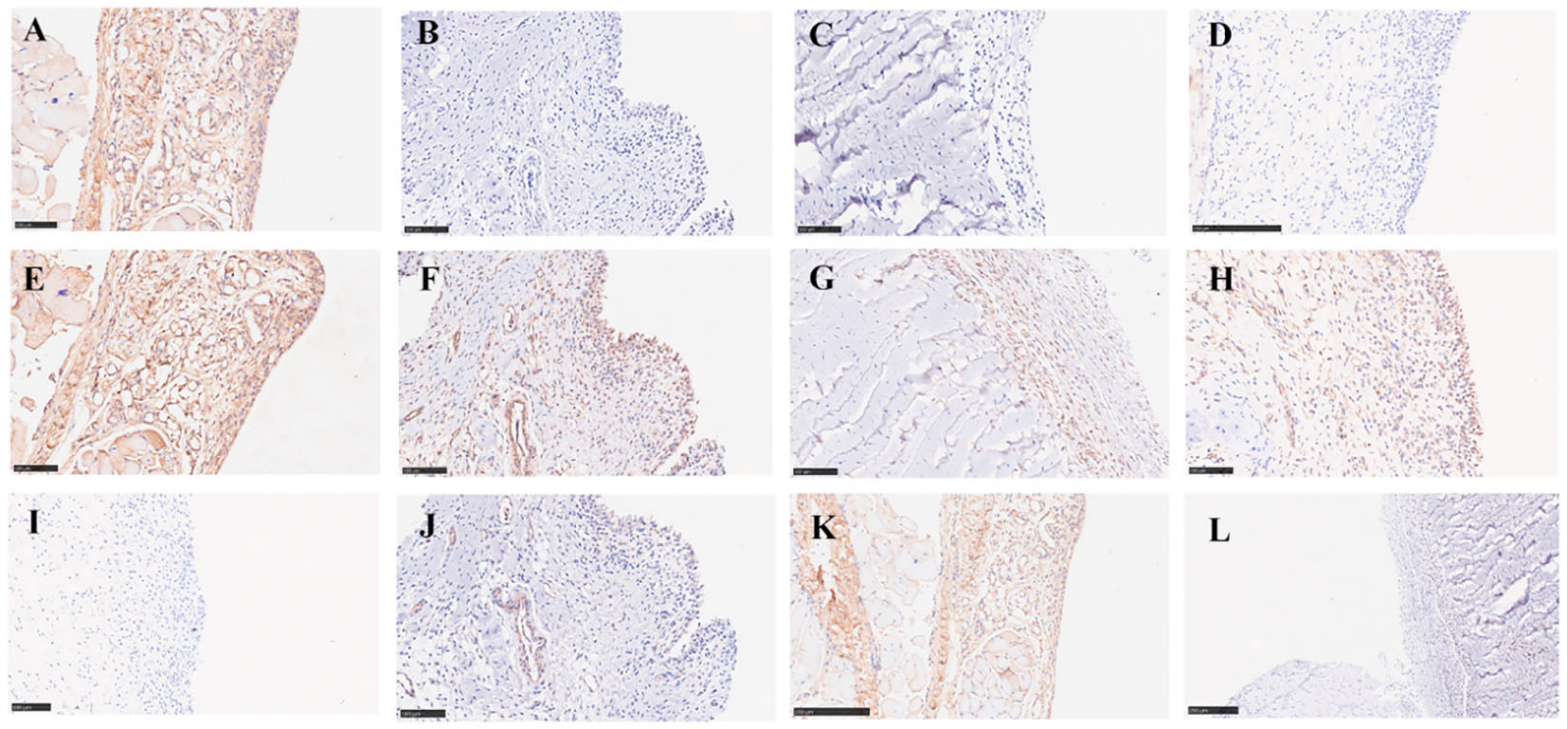
Impact of Resveratrol on TLR4, MyD88, and NF-κB Protein Expression in GA Model Rat Ankle Synovial Tissue. **(A)** TLR4 Expression in the Blank Control Group; **(B)** TLR4 Expression in the Model Group; **(C)** TLR4 Expression in the Positive Control Group; **(D)** TLR4 Expression in the Resveratrol Group; **(E)** MyD88 Expression in the Blank Control Group; **(F)** MyD88 Expression in the Model Group; **(G)** MyD88 Expression in the Positive Control Group; **(H)** MyD88 Expression in the Resveratrol Group; **(I)** NF-κB Expression in the Blank Control Group; **(J)** NF-κB Expression in the Model Group; **(K)** NF-κB Expression in the Positive Control Group; **(L)** NF-κB Expression in the Resveratrol Group.

Immunohistochemical results showed minimal positive expression of TLR4, MyD88, and NF-κB in the ankle synovial tissue of the normal control group rats. In contrast, there was a significant increase in positive expression in the ankle synovial tissue of the model group rats. The high-dose Resveratrol-treated group showed a significant decrease in positive expression compared to the model group (**Figures 4A–L**).

### 4.7 Molecular docking

**Figure 5:**
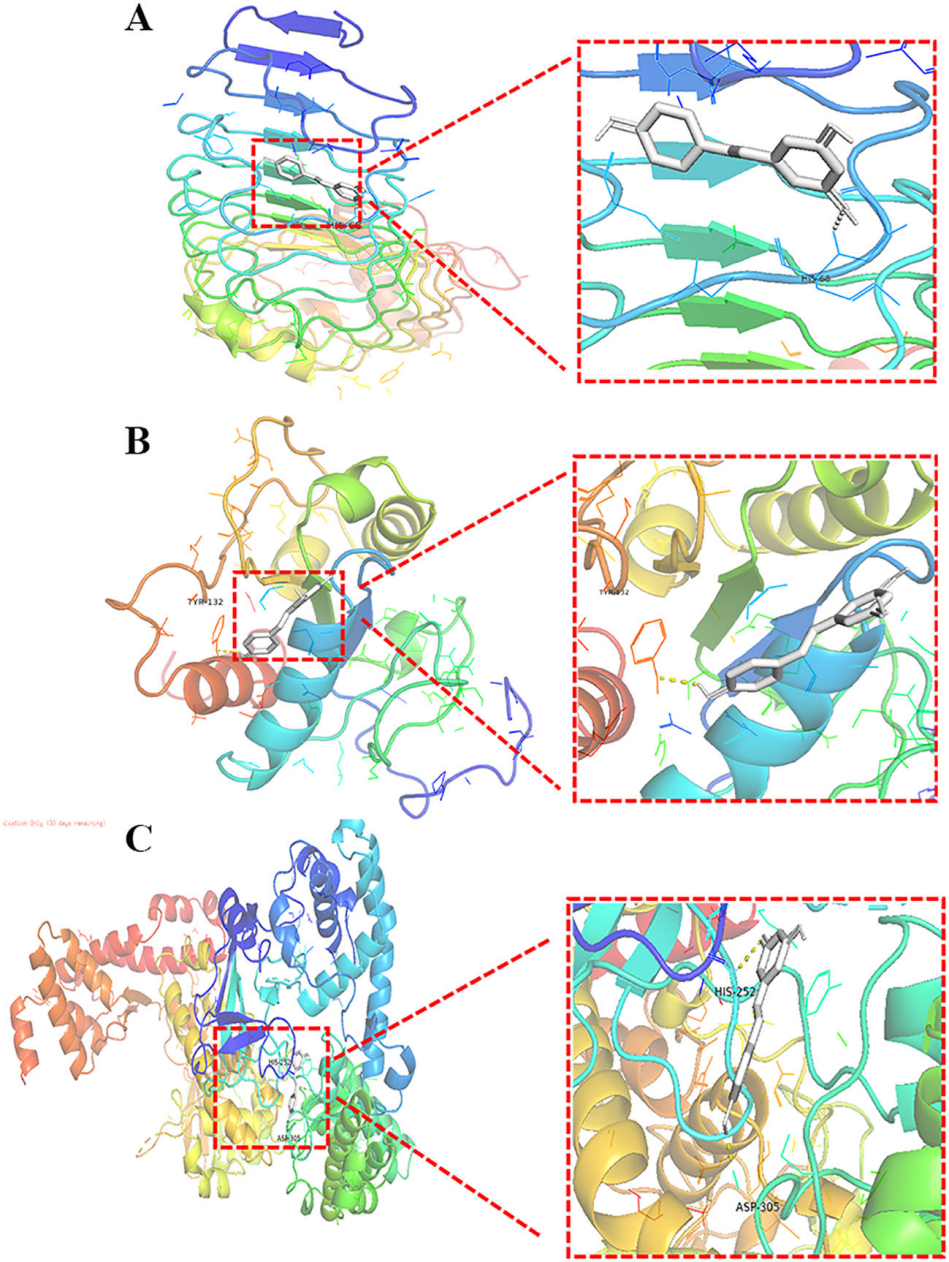
Molecular Docking of Resveratrol with TLR4, MyD88, and NF-κB. **(A)** Docking of Resveratrol with TLR4; **(B)** Docking of Resveratrol with MyD88; **(C)** Docking of Resveratrol with NF-κB.

Resveratrol showed good binding activity with the target proteins TLR4, MyD88, and NF-κB, with docking scores of -5.7, -5.9, and -7.6 kcal/mol, respectively, indicating a strong binding ability. Refer to (**Table 4**) and (**Figure 5**) for more details.

**Table 4:** Binding energies of resveratrol with TLR4, MyD88, NF-κB.

Target	TLR4	MyD88	NF-κB
Res	-5.7	-5.9	-7.6

### 4.8 Resveratrol delays the progression of gastric atrophy by regulating NF-κB, MAPK, and JAK2/STAT3 signaling pathways

**Figure 6:**
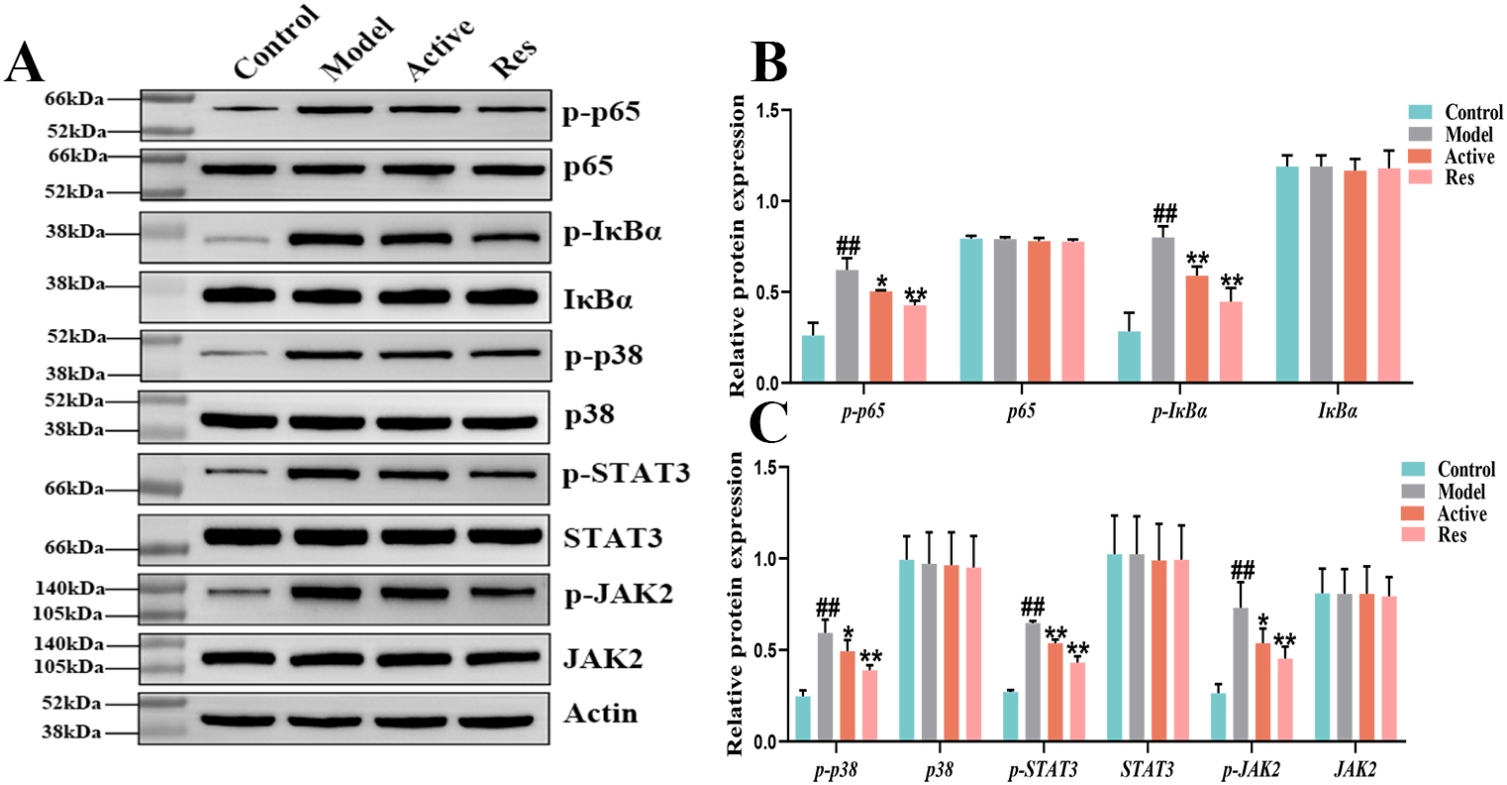
Effect of Res on NF-κB, MAPK and JAK2/STAT3 pathway proteins in GA ankle tissue. **(A)** Western blots of p65, p-p65, IκBα, p-IκBα,STAT3, p-STAT3, JAK2, and p-JAK2 in the ankle tissue; **(B, C)** Quantification of each western blotting band. Compared to the blank control group, #p<0.05, ##p<0.01; Compared to the model group, *p<0.05, **p<0.01.

To further elucidate the role of the NF-κB signaling pathway in the treatment of GA with Res, Western blot was used to detect the protein expression of NF-κB signaling pathway-related proteins p65, p-p65, IκBα, and p-IκBα.The results are shown in (**Figure 6**), during the progression of GA, the phosphorylation levels of p-p65 and p-IκBα were increased, indicating activation of the NF-κB signaling pathway. After intervention with resveratrol, the phosphorylation levels of p-p65 and p-IκBα were significantly reduced, further demonstrating that resveratrol delays the progression of GA by inhibiting the activation of the NF-κB signaling pathway. Meanwhile, we found that Res mainly intervenes in the progression of GA through the inflammatory signaling pathway. In order to further investigate the mechanism of action of resveratrol in the treatment of GA, we also selected the inflammatory signaling pathways MAPK, JAK2/STAT3, and explored the roles of these two pathways in the treatment of GA with Res. We detected the protein expression levels of p38, p-p38 in the MAPK signaling pathway, as well as STAT3, p-STAT3, JAK2, and p-JAK2. The results are shown in (**Figure 6**), during the progression of GA, the phosphorylation levels of p-p38, p-STAT3, and p-JAK2 were increased, indicating activation of the MAPK and JAK2/STAT3 signaling pathways. However, Res intervention could significantly reverse this phenomenon and delay the progression of the disease. This further illustrates that Res can exert anti-inflammatory effects through multiple signaling pathways, thereby achieving the preventive and therapeutic effects on the disease, as shown in the **Supplementary Figure S1.**

### 4.9 Analysis of rat serum metabolism

**Figure 7:**
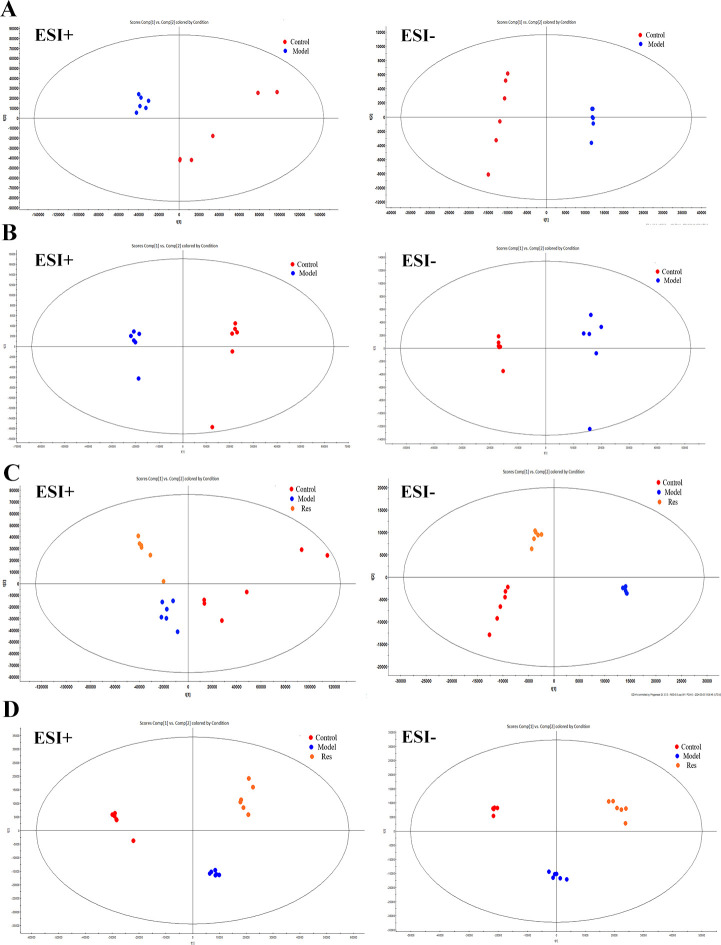
Impact of Resveratrol on Serum and ankle Metabolic Profile in GA Model Rats. **(A)** PCA plot of serum metabolism in blank and model groups in positive and negative ion modes; **(B)** PCA plot of ankle metabolism in blank and model groups in positive and negative ion modes; **(C)** PCA plot of serum metabolism in blank,model and Resveratrol group in positive and negative ion modes; **(D)** PCA plot of ankle metabolism in blank,model and Resveratrol group in positive and negative ion modes).

**Figure 8:**
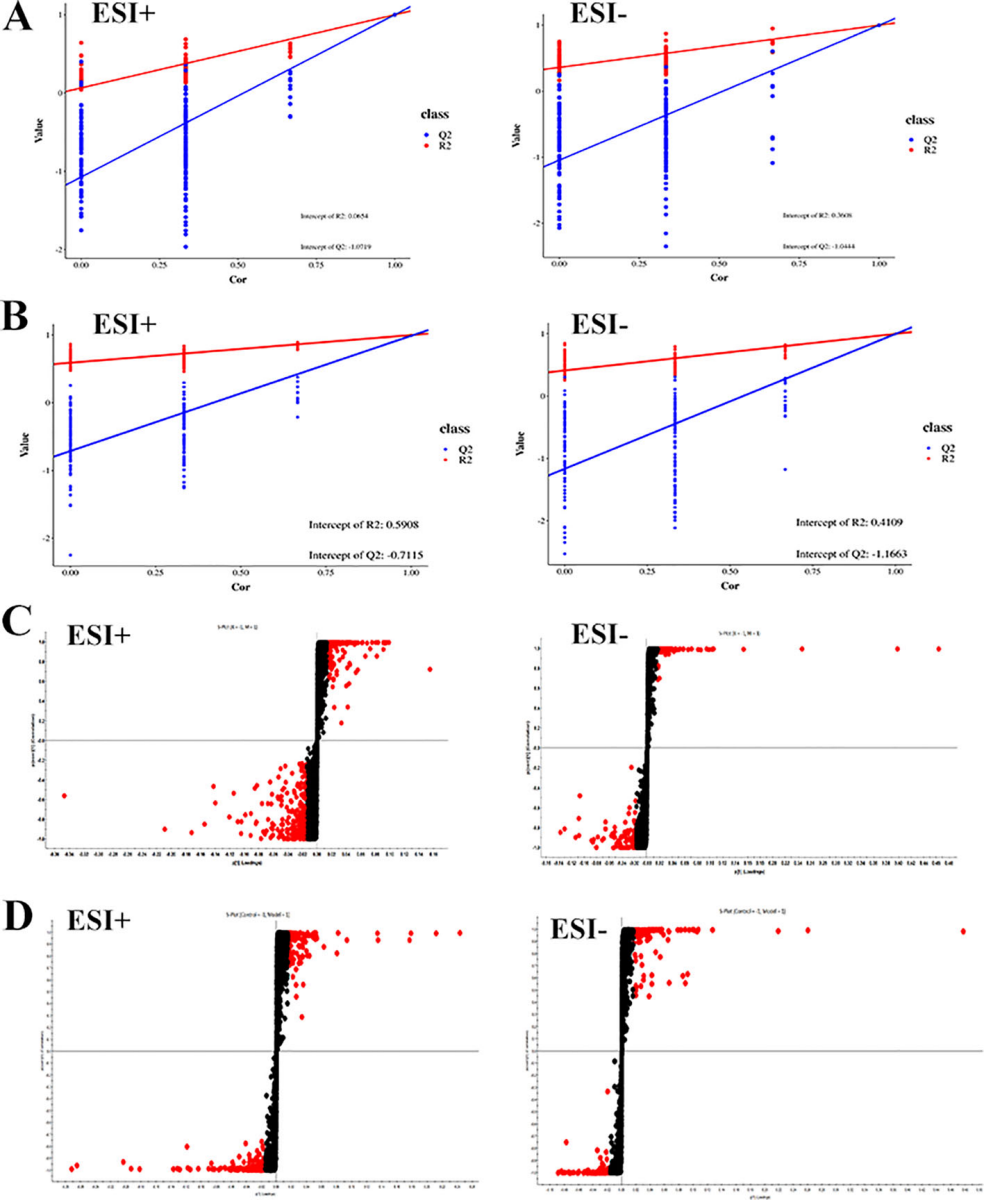
Impact of Resveratrol on Serum and ankle Metabolic Profile in GA Model Rats. **(A)** Quality control plots of the serum metabolism in the positive-negative ion mode; **(B)** Quality control plots of the ankle metabolism in the positive-negative ion mode; **(C)** S-Plot of the serum metabolism in the positive-negative ion mode; **(D)** S-Plot of the ankle metabolism in the positive-negative ion mode).

By analyzing the data generated by UPLC-TOF-MS, the unsupervised orthogonal partial least squares discriminant analysis method was applied to process PCA, to represent the metabolic profiles of serum and ankle joint tissue samples of GA rats. The results showed that both in serum samples and ankle joint tissue samples, the blank group and the model group were clearly separated, and there were significant differences between the two groups, reflecting the high reliability of the model established in this study, as shown in (**Figures 7A, B**); when comparing the blank group, model group, and Res treatment group, it can be found that all three groups showed their own clustering and the Res group showed a trend towards normal treatment group, indicating that Res can improve the metabolic deviations in serum and ankle joint tissue samples of model rats, as shown in (**Figures 7C, D**). In order to further verify the separation of samples between the blank control group and the model group, and to maximize the inter-group separation effect, the OPLS-DA method was used for supervised analysis. The model established by this supervised method had a high explanatory rate and prediction rate. As shown in (**Figures 8A, B**), OPLS-DA analysis significantly improved the separation and clustering ability of the data, and the metabolic spectra of the two groups of samples showed their own clustering, located in three different regions of the scatter plot scores, with good similarity and little overlap between the metabolic trajectories of each group, indicating significant biological changes in the serum and ankle joint tissue of GA rats. The stability and prediction ability of the OPLS-DA model should be evaluated through cross-validation (CV) and alternative experiments. The score plot model underwent 100 random permutation tests, and the R2 and Q2 generated by any random permutation were both less than the R2 and Q2 of the model group, indicating that the established model was reliable, did not overfit, and had good predictive ability. By observing the S-plot of serum and ankle joint tissue samples, it can be seen that most of the metabolite ions clustered around the origin, with only a few ions deviating from the origin, indicating differences between the two groups, as shown in (**Figures 8C, D**).

By predicting the changes in metabolites in rat serum and ankle joint tissue samples, metabolites the criteria of VIP>1, fold change>1.2 or <0.8, and q-value>0.5 were identified. A total of 24 differential metabolites were identified in the serum, and 22 differential metabolites were identified in the ankle joint tissue. After administration of resveratrol, the levels of 5 biomarkers were significantly downregulated, while the levels of 19 biomarkers were significantly upregulated, as shown in (**Tables 5**, **6**).

**Table 5:** Common potential biomarkers in serum samples of GA model rats in positive and negative ion modes after resveratrol intervention.

NO.	Rt min	M/ Z	Ion mode	Proposed Composition	HMDB	Postulated Identity	Trend	
							Control to Model	Model to Res
1	0.90	310.1289	Pos	C37H56O3	HMDB0012148	2-Hexaprenyl-6-methoxy-1,4-benzoquinol	↑^##^	↓**
2	4.49	508.3758	Pos	C26H54NO6P	HMDB0013122	LysoPC(P-18:0)	↓^#^	↑*
3	3.54	480.3447	Pos	C24H50NO6P	HMDB0010407	LysoPC(P-16:0)	↓^##^	↑**
4	5.02	358.3681	Pos	C22H44O2	HMDB0000944	Behenic acid	↑^##^	↓**
5	0.60	216.0978	Pos	C8H10N2O4	HMDB0015188	Mimosine	↑^##^	↓**
6	3.23	346.3317961	Pos	C20H40O3	HMDB0032477	Polyoxyethylene 40 monostearate	↑^##^	↓**
7	2.76	362.3267	Pos	C20H40O4	HMDB0031923	10,20-Dihydroxyeicosanoic acid	↓^##^	↑**
8	6.65	284.2952	Pos	C18H34O	HMDB0030965	9-Octadecenal	↑^##^	↓**
9	2.75	290.2693	Pos	C16H32O3	HMDB0006294	16-Hydroxyhexadecanoic acid	↓^##^	↑**
10	3.25	318.3005	Pos	C18H39NO3	HMDB0004610	Phytosphingosine	↓^#^	↑*
11	1.03	122.0969	Pos	C8H11N	HMDB0002017	1-Phenylethylamine	↓^##^	↑*
12	8.39	838.5982599	Neg	C46H84NO7P	HMDB0008489	PC( 20 :4( 8Z,11Z,14Z,17Z) /P-18: 0 )	↓^##^	↑**
13	7.43	831.6604485	Neg	C45H91N2O6P	HMDB0012103	SM( d18 : 1/22 : 0 )	↓^##^	↑**
14	3.80	550.3517	Neg	C26H52NO6P	HMDB0010408	LysoPC(P-18:1(9Z))	↓^##^	↑**
15	5.08	568.3627	Neg	C26H54NO7P	HMDB0010384	LysoPC(18:0)	↓^##^	↑**
16	3.98	540.3308	Neg	C24H50NO7P	HMDB0010382	LysoPC(16:0)	↓^##^	↑*
17	6.98	747.5677	Neg	C39H79N2O6P	HMDB0010169	SM( d18 : 1/16 : 0 )	↓^##^	↑**
18	5.23	594.3774	Neg	C28H56NO7P	HMDB0010391	LysoPC(20:1(11Z))	↓^##^	↑**
19	3.98	480.3089	Neg	C23H48NO7P	HMDB0010381	LysoPC(15:0)	↓^#^	↑**
20	8.51	719.5361	Neg	C37H75N2O6P	HMDB0012097	SM( d18 : 1/14 : 0 )	↓^##^	↑**
21	5.08	508.3401	Neg	C25H52NO7P	HMDB0012108	LysoPC(17:0)	↓^#^	↑**
22	5.34	554.3827951	Neg	C26H56NO6P	HMDB0011149	LysoPC(O-18:0)	↓^##^	↑*
23	4.22	436.2827	Neg	C21H44NO6P	HMDB0011152	PE( P-16 : 0e/0 : 0 )	↓^##^	↑**
24	3.53	540.3305	Neg	C24H50NO7P	HMDB0240262	LysoPC(0:0/16:0)	↓^##^	↑*

Compared to the blank control group, ^#^ *p*<0.05, ^##^ *p*<0.01; Compared to the model group, *p<0.05, **p<0.01. ↓, represents a decrease in metabolite content; ↑, represents an increase in metabolite content.

**Table 6:** Common potential biomarkers in ankle tissue samples of GA model rats in positive and negative ion modes after resveratrol intervention.

NO.	Rt min	M/ Z	Ion mode	Proposed Composition	HMDB	Postulated Identity	Trend	
							Control to Model	Model to Res
**1**	397.2701	8.51	pos	C24H38O3	HMDB0304752	3E-tetradecenoic acid	↑^#^	↓*
**2**	357.2779	8.83	pos	C24H36O2	HMDB0001182	6,8-Dihydroxypurine	↑^#^	↓*
**3**	277.2149	9.94	pos	C18H28O2	HMDB0244507	13-Docosenamide	↓^##^	↑**
**4**	166.0888	3.52	pos	C9H13NO3	HMDB0000269	Sphinganine	↓^#^	↑*
**5**	167.0391	3.91	pos	C5H4N4O3	HMDB0034181	3-Methylcyclopentadecanone	↓^##^	↑**
**6**	237.0411	4.95	pos	C6H8O7	HMDB0242058	N-Myristoyl Proline	↓^##^	↑**
**7**	188.0702296	1.00375	pos	C11H9NO2	HMDB0000734	Indoleacrylic acid	↓^##^	↑**
**8**	357.2779	8.83	pos	C24H36O2	HMDB0252076	Tetracosahexaenoic acid	↑^#^	↓*
**9**	241.1072n	1.18	pos	C10H15N3O4	HMDB0002224	5-Methyldeoxycytidine	↓^##^	↑**
**10**	219.1145n	2.04	pos	C9H17NO5	HMDB0000210	Pantothenic acid	↓^##^	↑**
**11**	174.0575	2.86	pos	C5H4N2O4	HMDB0000226	Orotic acid	↓^##^	↑**
**12**	163.0697n	4.32	pos	C9H9NO2	HMDB0004186	3-Methyldioxyindole	↓^##^	↑**
**13**	260.041092	0.83955	neg	C9H11NO8	HMDB0260161	(4S)-4-Amino-1,3-dioxohexane-1,2,6-tricarboxylic acid	↑^#^	↓*
**14**	403.1625422	6.583283333	neg	C9H14O5	HMDB0033092	3-(1-Hydroxymethyl-1-propenyl)pentanedioic acid	↓^##^	↑**
**15**	791.5074596	8.9073	neg	C39H71O11P	HMDB0270684	PG(a-15:0/18:2(10E,12Z)+=O(9))	↑^#^	↓*
**16**	795.4835879	8.800183333	neg	C43H73O11P	HMDB0271447	PG(22:5(4Z,7Z,10Z,13Z,19Z)-O(16,17)/i-15:0)	↑^#^	↓*
**17**	635.3924695	8.9073	neg	C31H59O8P	HMDB0114795	PA(14:1(9Z)/14:0)	↓^##^	↑**
**18**	167.0391	3.91	neg	C5H4N4O3	HMDB0000289	Uric acid	↑#	↓*
**19**	163.0405	4.09	neg	C9H8O3	HMDB0000205	Phenylpyruvic acid	↑^#^	↓*
**20**	308.12	4.39	neg	C9H17N3O6	HMDB0240254	Creatine riboside	↑^#^	↓*
**21**	237.0411	4.95	neg	C6H8O7	HMDB0000094	Citric acid	↓^#^	↑*
**22**	267.0933	5.2	neg	C10H12N4O5	HMDB0000195	Inosine	↓^##^	↑**

Compared to the blank control group, ^#^ *p*<0.05, ^##^ *p*<0.01; Compared to the model group, *p<0.05, **p<0.01. ↓, represents a decrease in metabolite content; ↑, represents an increase in metabolite content.

### 4.10 Pathway analysis of potential biomarkers in rat serum samples

Enrich the screened biomarkers, introduce the screened differential metabolite group into MetaboAnalyst for differential metabolic pathway enrichment analysis, and use metabolic pathways with a critical value greater than 0.1 as potential key metabolic pathways. We found that serum metabolism mainly enriched in Sphingolipid metabolism, Glycerophospholipid metabolism, Linoleic acid metabolism, Alpha-Linolenic acid metabolism, and other six metabolic pathways; ankle joint tissue metabolism mainly enriched in Phenylalanine, tyrosine and tryptophan biosynthesis, Phenylalanine metabolism, Purine metabolism, Pantothenate and CoA biosynthesis, and other nine metabolic pathways, as shown in (**Tables 7**, **8**). After drug intervention, the phenomenon of metabolic pathway disorder was significantly improved, and the content of metabolites showed a trend of regression, indicating that resveratrol plays a therapeutic role in GA by regulating the relative levels of metabolic biomarkers and modulating abnormal metabolic key pathways. The metabolic schematic diagram is shown in the figure.

**Table 7:** MetPA analyses of resveratrol-regulated metabolic pathways in serum.

pathway name	Total	Expected	Hits	Raw p	-LOG	Holm adjust	FDR	Impact
Sphingolipid metabolism	21	0.067742	2	0.0017067	2.7678	0.14337	0.14337	0.00406
Glycerophospholipid metabolism	36	0.11613	2	0.0050211	2.2992	0.41675	0.21088	0.11182
Linoleic acid metabolism	5	0.016129	1	0.016046	1.7946	1	0.44929	0
Alpha-Linolenic acid metabolism	13	0.041935	1	0.04129	1.3842	1	0.8671	0
Ether lipid metabolism	20	0.064516	1	0.062952	1.201	1	1	0.14458
Arachidonic acid metabolism	36	0.11613	1	0.111	0.9547	1	1	0

**Table 8:** MetPA analyses of resveratrol-regulated metabolic pathways in ankle.

pathway name	Total	Expected	Hits	Raw p	-LOG	Holm adjust	FDR	Impact
Phenylalanine, tyrosine and tryptophan biosynthesis	4	0.017778	1	0.017676	1.7526	1	0.94356	0
Phenylalanine metabolism	8	0.035556	1	0.035084	1.4549	1	0.94356	0.2619
Purine metabolism	70	0.31111	2	0.035384	1.4512	1	0.94356	0.00245
Pantothenate and CoA biosynthesis	20	0.088889	1	0.085731	1.0669	1	1	0.0068
Citrate cycle (TCA cycle)	20	0.088889	1	0.085731	1.0669	1	1	0.09038
Alanine, aspartate and glutamate metabolism	28	0.12444	1	0.11821	0.92733	1	1	0
Glyoxylate and dicarboxylate metabolism	32	0.14222	1	0.13408	0.87263	1	1	0.03175
Sphingolipid metabolism	32	0.14222	1	0.13408	0.87263	1	1	0.07838
Pyrimidine metabolism	39	0.17333	1	0.16126	0.79247	1	1	0.04697

## 5 Conclusion

The NF-κB signaling pathway is recognized to be closely related to the pathogenesis and inflammatory manifestations of gout arthritis (GA). Studies have shown that when serum uric acid levels increase in GA rats, oversaturated urate salts deposit, forming crystals and precipitates in synovial tissues. Uric acid sodium crystals are recognized as danger signals by Toll-like receptors (TLRs), activating TLRs that recruit myeloid differentiation factor 88 (MyD88). Subsequently, interleukin-1 receptor-associated kinase (IRAK) and transforming growth factor-β-activated kinase 1 (TAK1) are activated, triggering the IκB kinase cascade reaction, activating the nuclear transcription factor NF-κB, exposing the p65 site, leading to translocation of NF-κB dimers to the nucleus, thereby initiating the expression of cytokines such as IL-1β, TNF-α related to inflammation and immunity, ultimately producing large amounts of inflammatory cytokines such as IL-1β, TNF-α, leading to a large number of neutrophils entering the joint site, promoting the generation and development of GA inflammation. This study found that in the MSU-induced GA rat model, serum biochemical indicators and levels of inflammatory factors were significantly increased, accompanied by severe synovial inflammation and marked neutrophil infiltration. The expression of TLR4, MyD88, NF-κB proteins in joint tissues increased, and the phosphorylation levels of p-p65 and p-IκB significantly increased. After resveratrol (Res) intervention, the pathological damage, uric acid, creatinine, blood urea nitrogen levels, and serum IL-1β, IL-8, TNF-α levels in the model rats were significantly reduced, as well as the expression of TLR4, MyD88, NF-κB proteins, and phosphorylation levels of p-p65 and p-IκB. These results suggest that Res delays disease progression by inhibiting the NF-κB signaling pathway.

The “TLR4/MyD88/NF-κB” signaling pathway is recognized to be closely associated with the pathogenesis and inflammatory manifestations of GA. Studies have shown that when the serum uric acid levels increase in GA rats, oversaturated urate deposits in the synovial tissues form crystals and precipitates (23). Uric acid sodium crystals, as danger signals, are recognized by TLRs, leading to the recruitment of myeloid differentiation factor 88 (MyD88) and subsequent activation of interleukin-1 receptor-associated kinases (IRAK) and transforming growth factor-beta activated kinase (TGF-β TAK1). This eventually triggers IκB kinase cascade reactions and activates the nuclear transcription factor NF-κB, leading to the expression of inflammatory cytokines such as IL-1β and TNF-α, which are responsible for the production of large amounts of IL-1β, TNF-α, and other inflammatory cytokines (24). Mature IL-1β is considered to be the initiating factor of inflammation in GA. It activates IL-1 receptors to exert the role of chemokines and other inflammatory regulatory factors, resulting in a large number of neutrophils entering the joint site, promoting the occurrence and development of GA (25). In addition, IL-1β can activate osteoclasts to differentiate and act on neurons, triggering excessive sensitivity to pain caused by inflammation. TNF-α can promote the secretion of cytokines such as IL-1β, IL-8, and PGE2 and the appearance of phagocytic activity of neutrophils, as well as the release of oxygen free radicals and proteinases, leading to congestion, edema, and infiltration of inflammatory cells at urate deposition sites, which further aggravate the condition (26). The results of this study showed that the levels of serum biochemical indicators and inflammatory factors were significantly increased in the MSU-induced GA rat model, accompanied by severe synovial inflammation and significant neutrophil infiltration. The protein expression levels of TLR4, MyD88, and NF-κB were elevated, and Res intervention significantly reduced the pathological damage, as well as the levels of uric acid, creatinine, blood urea nitrogen, serum IL-1β, IL-8, TNF-α, and the protein expression levels of TLR4, MyD88, and NF-κB in the model rats. In addition, Res significantly inhibited the proliferation of THP-1 cells induced by MSU and reduced the levels of inflammatory cytokines in the cell culture supernatant, as well as the protein expression levels of TLR4, MyD88, and NF-κB. Res can inhibit the activation of NF-κB by weakening the recognition and internalization of TLR4 in the inflammatory response, blocking the intracellular transduction at the connection with MyD88, and subsequently slowing down the inflammatory response.

Meanwhile, in order to further explore the mechanism of Res in preventing GA, we investigated its mechanism from the perspective of MAPK and JAK/STAT3 signaling pathways. MAPK is a serine-threonine protein kinase family, including p-JNK/JNK, p-ERK/ERK, and p-P38/P38, which can regulate various cellular activities, including cell proliferation, differentiation, apoptosis, inflammation, and innate immunity. MAPK signaling can be activated by pro-inflammatory cytokines, thereby participating in the stimulation response of inflammatory cytokines in the process of chronic inflammation. Relevant studies have shown that activation of TLR4 can activate the MAPKs signaling pathway, thereby regulating the release and expression of inflammatory cytokines, exacerbating the disease progression of gout and its complications (27). The Janus kinase/signal transducer and activator of transcription (JAK/STAT) signaling pathway is an important intracellular signaling pathway discovered in recent years, involved in the transduction of growth factors and cytokines across cell membranes, crucial for initiating innate immunity and coordinating adaptive immunity. It plays a significant role in the pathogenesis of gouty arthritis. Research has shown that activation of the pro-inflammatory cytokine IL-6 leads to sustained activation of the JAK/STAT signaling pathway, which can further elevate the levels of pro-inflammatory cytokines such as TNF-α, IL-1β, IL-6, IL-8, IL-12, and IL-18. Reducing the phosphorylation levels of JAK2/STAT3 can help repair joint damage (28). Reduced JAK/STAT signaling cascade can decrease the deposition of monosodium urate crystals and lower the serum urate levels, effectively inhibiting joint pain and inflammation (29). The results of this study further demonstrate that the phosphorylation levels of p38, p-JAK2, and p-STAT3 in the ankle joint tissues of GA model rats are elevated, indicating activation of the JAK/STAT3 signaling pathway. After Res intervention, the phosphorylation levels of p38, p-JAK2, and p-STAT3 are significantly reduced, inhibiting the activation of the JAK/STAT3 signaling pathway and delaying the progression of the disease. Additionally, related studies have shown that inhibiting the phosphorylation of JAK2/STAT3 can suppress the activation of NF-κB (30). Resveratrol inhibits the activation of the TLR4/MyD88/NF-κB signaling pathway to suppress the MAPK and JAK/STAT3 signaling pathways, thereby achieving the preventive and therapeutic effects on diseases. Please refer to the specific pathway diagram in the **Supplementary Figure S2.**

Glycerophospholipid metabolism: Phospholipids mainly include glycerophospholipids and sphingolipids, among which glycerophospholipids are components of bile and cell membranes, participating in the formation of biological membranes, membrane recognition, and signal transduction. They can eventually be hydrolyzed into free fatty acids and lysophospholipids (31). Studies have found significant differences in glycerophospholipid metabolism between gout patients and control groups through metabolomics analysis of urine samples (32). More and more animal experiments have discovered lipid metabolism disorders in high uric acid or gout rats, with glycerophospholipid metabolism pathway being the most affected (33, 34). In this study, Res was found to attenuate the levels of certain glycerophospholipid metabolites, including lyso-phosphatidylcholines [LysoPC(P-18:0), LysoPC(P-16:0), LysoPC(P-18:1(9Z)), LysoPC(18:0), LysoPC(16:0), LysoPC(20:1(11Z)), LysoPC(15:0), LysoPC(17:0), LysoPC(O-18:0), LysoPC(0:0/16:0)], phosphatidylcholine [PC(20:4(8Z,11Z,14Z,17Z)/P-18:0)], and phosphatidylethanolamine [PE(P-16:0e/0:0)]. This suggests that Res may improve glycerophospholipid metabolism disorders caused by GA by promoting the transformation and metabolism of lipid metabolites.

Phospholipid metabolism: Sphingolipids are a group of lipids containing sphingosine molecules, including sphingomyelin, sphingosine, and 1-phosphorylated sphingosine, etc. (35, 36). A longitudinal cohort study found that primary hypertension combined with hyperuricemia is related to sphingolipid metabolism disorder (33).Our metabolomics analysis found that levels of sphingomyelin (SM(d18:1/22:0), SM(d18:1/16:0), SM(d18:1/14:0)) and sphingosine were downregulated in serum samples of gouty arthritis model, and sphingosine content in ankle joint tissue samples was also significantly decreased. After Res intervention, all these levels were significantly restored. This indicates that Res can improve the sphingolipid metabolism disorder in serum and synovial fluid during the progression of gouty arthritis.

Arachidonic acid metabolism: Metabolites of arachidonic acid can inhibit inflammation induced by leukotrienes, interleukin 2, prostaglandin E2, and histamine (37). They also increase vascular permeability and exudate production, and cause leukocyte accumulation in inflamed area (38). This study found that the expression of 1-phenylethylamine, a metabolite of arachidonic acid metabolism, was significantly lower in the GA model group compared to the control group, and the Res group had significantly higher expression compared to the GA group. This indicates that Res may improve arachidonic acid metabolism disorder in GA by regulating 1-phenylethylamine metabolism. Additionally, related research has shown that arachidonic acid can inhibit inflammatory responses by inhibiting the NF-κB signaling pathway, further preventing acute inflammatory responses in GA (39).

Amino acid metabolism plays a crucial role in adaptive and innate immunity, regulating the activation of immune cells and antibody production (12). Phenylalanine, tyrosine, and tryptophan are three aromatic amino acids involved in biosynthesis, sharing a common pathway starting from phosphoenolpyruvate and erythrose-4-phosphate to form branched acids, which then synthesize these three amino acids (40). Tryptophan can be metabolized into proteins and a series of biologically active molecules, reflecting the diverse physiological processes controlled by it (41). Studies have shown (42) that tryptophan metabolism disorders exist in gout and its complications. In the metabolic process, tryptophan may be converted to acetyl-CoA, entering the tricarboxylic acid cycle, producing alpha-ketoglutarate, phenylpyruvate, and indoleacetic acid. Hydroxylation of phenylalanine in the liver can generate tyrosine, and phenylpyruvate is an intermediate product in the synthesis of the three amino acids (43). This study indicates a significant increase in phenylpyruvate after modeling, and resveratrol may affect the synthesis and degradation of aromatic amino acids by regulating phenylpyruvate, thereby regulating the biosynthesis of phenylalanine, tyrosine, and tryptophan for disease prevention and treatment. Additionally, the study shows that the phenylalanine metabolic pathway affects the biosynthesis pathways of pantothenic acid and coenzyme A. Pantothenic acid is a precursor of coenzyme A, which is a cofactor in various metabolic reactions, and its synthesis is regulated by acetyl-CoA synthetase. Coenzyme A plays a crucial role in energy metabolism and is involved in the metabolism of glucose, proteins, and lipids through the TCA cycle (44). The results of this study show that resveratrol can regulate the content of pantothenic acid in the joint dialysate of GA rats; meanwhile, citric acid, as a key intermediate product of the tricarboxylic acid cycle, not only participates in the aerobic oxidation of glucose but also is a major pathway in amino acid metabolism (45). Furthermore, the study results indicate a significant decrease in citric acid content in the joint dialysate of GA model rats, suggesting disorders in the biosynthesis pathways of pantothenic acid and coenzyme A, as well as the TCA cycle, which are significantly reversed after resveratrol intervention.

Research suggests that MSU may trigger mitochondrial dysfunction and lead to an increase in mitochondrial ROS, thereby inducing the occurrence of the cell necroptosis pathway. The TCA cycle is the center of energy metabolism and is closely related to mitochondrial function. In this case, the increase in mitochondrial fatty acid oxidation can induce an increase in acetyl-CoA production, which amplifies the formation of more ROS, enhancing a vicious cycle of inflammasome activation (46, 47). The results of this study show that the ROS levels in GA model rats are significantly elevated, and ROS can further activate the NF-KB signaling pathway, thereby inducing the secretion of inflammasomes. Meanwhile, it can induce an increase in acetyl-CoA and abnormalities in the TCA cycle, further activating inflammasomes. This suggests that Res may regulate the biosynthesis of phenylalanine, tyrosine, and tryptophan, pantothenic acid, and coenzyme A biosynthesis pathways, TCA cycle metabolic pathways, and regulate the NF-KB signaling pathway, thereby delaying disease progression.

In summary, resveratrol can reduce arthritis inflammation in GA rats, and its mechanism may involve regulating the biosynthesis of arachidonic acid, phenylalanine, tyrosine, and tryptophan, pantothenic acid, and coenzyme A biosynthesis pathways, TCA cycle, and other metabolic pathways to regulate NF-κB, MAPK, JAK/STAT3 signaling pathways, thereby inhibiting acute inflammatory responses during GA attacks. However, in the NLRP3-dependent cell pyroptosis pathway, TLR4 is the classical activation pathway. Therefore, we speculate that the mechanism of resveratrol in anti-gouty arthritis may involve inhibiting the NLRP3-mediated TLR4/MyD88/NF-κB pathway by regulating relevant metabolic pathways, thereby inhibiting pyroptosis and slowing down the inflammatory response. However, further experiments are needed to verify whether it inhibits pyroptosis and whether pyroptosis occurs in the MSU-induced GA model, which is also the focus of our future research.

## Data Availability

The raw data supporting the conclusions of this article will be made available by the authors, without undue reservation.

## Supplementary material

Supplementary Figure S1 Metabolic pathway schematic

Supplementary Figure S2 Proposed mechanism for Res to treat GA
