# Corrigendum: Investigating the mechanisms of resveratrol in the treatment of gouty arthritis through the integration of network pharmacology and metabolics

**DOI:** 10.3389/fendo.2025.1551182

**Published:** 2025-02-06

**Authors:** Xiaomin Xu, Donghua Yu, Yu Wang, Xin Jiang, Fang Lu, Shumin Liu

**Affiliations:** Research Institute of Traditional Chinese Medicine, Heilongjiang University of Chinese Medicine, Harbin, China

**Keywords:** metabonomics, network pharmacology, gouty arthritis, resveratrol, NF- kappa B, MAPK, stat3, JAK2

In the published article, there was an error in the author list, and author Donghua Yu was erroneously excluded Co-first author. The corrected author list appears below.

“Xiaomin Xu^†^, Donghua Yu^†^, Yu Wang, Xin Jiang, Fang Lu^*^, Shumin Liu^*^”

The authors apologize for this error and state that this does not change the scientific conclusions of the article in any way. The original article has been updated.

