# Supplementary figures and images for: Investigating the mechanisms of resveratrol in the treatment of gouty arthritis through the integration of network pharmacology and metabolics
Source: Front Endocrinol (Lausanne). 2024 Oct 29;15:1438405. doi: 10.3389/fendo.2024.1438405 (PMC11555470; doi:10.3389/fendo.2024.1438405)

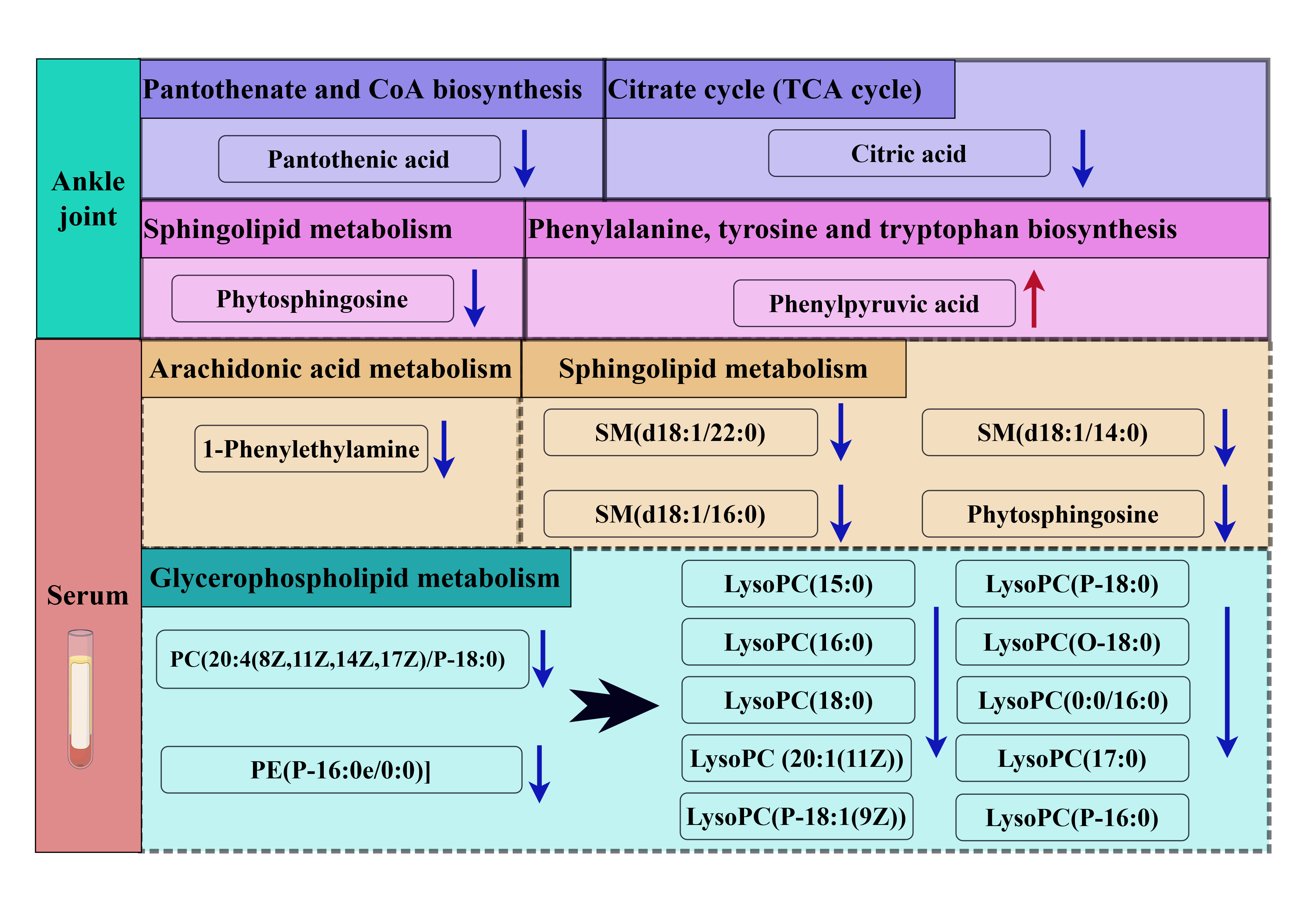

Supplement: Supplementary Figure S1 — Metabolic pathway schematic [file Image1.tif]

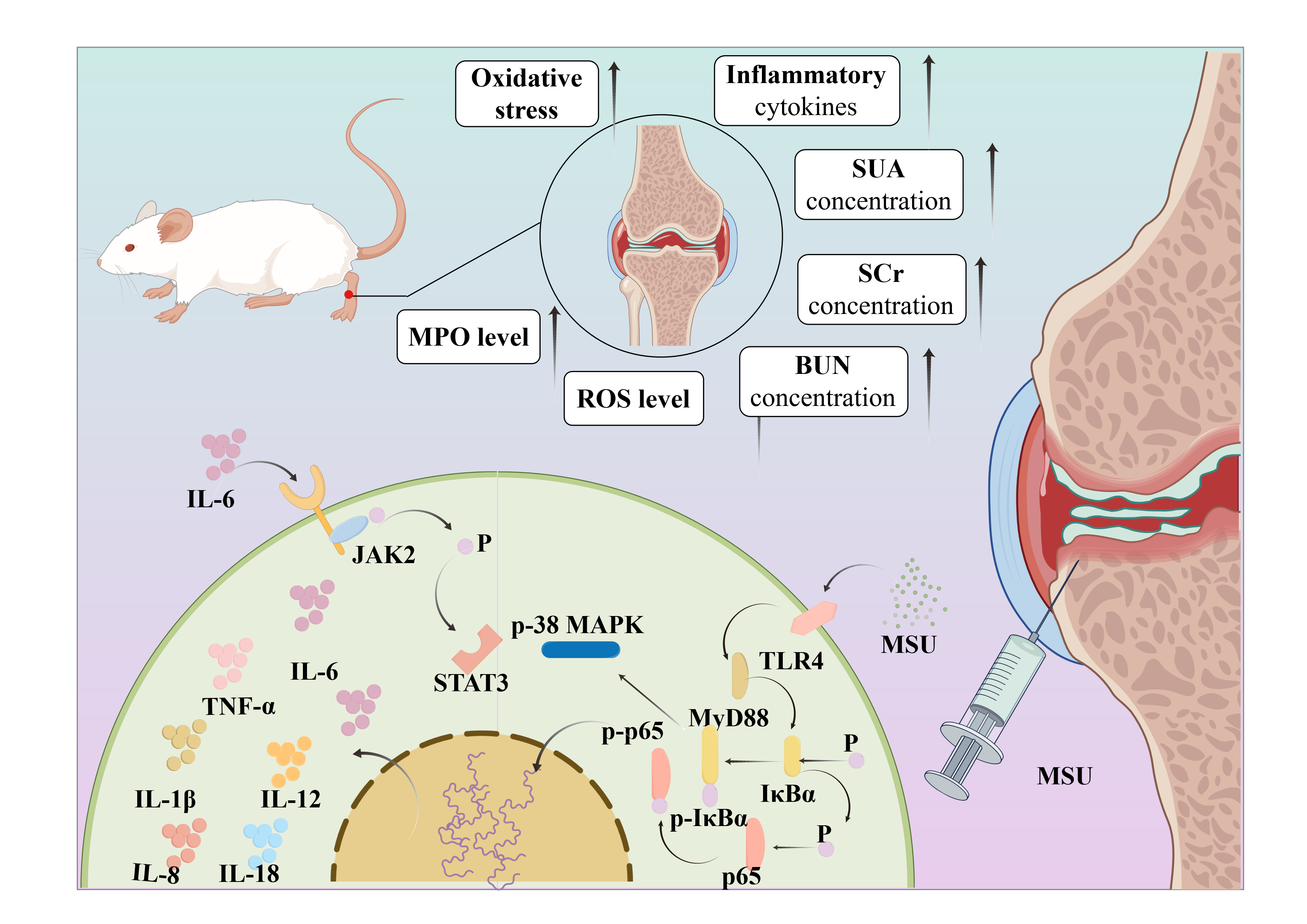

Supplement: Supplementary Figure S2 — Proposed mechanism for Res to treat GA [file Image2.tif]
